# Sleep Disturbances and Circadian Phase Delay in People Living with Human Immunodeficiency Virus (HIV): A Systematic Review

**DOI:** 10.3390/ijms27209189

**Published:** 2026-10-16

**Authors:** Antoni Gomila-Cardona, Saúl Ferrández-Sempere, Francesca Cañellas, Antoni Gamundí, Mourad Akaârir, Cristina Nicolau, José Ángel Rubiño-Díaz

**Affiliations:** 1 Department of Biology, University of the Balearic Islands (UIB), 07122 Palma, Spain; 2 Health Research Institute of the Balearic Islands (IdISBa), 07010 Palma, Spain; 3 Son Espases University Hospital (HUSE), 07010 Palma, Spain; 4 Nursing School, CESAG-UP Comillas (University College Alberta Giménez affiliated to Comillas Pontifical University), 07013 Palma, Spain

**Keywords:** circadian rhythms, HIV, human immunodeficiency virus, people living with HIV, sleep quality, sleep wake disorders

## Abstract

Advances in HIV research and antiretroviral therapy (ART) have substantially increased life expectancy among people living with HIV (PLHIV). However, challenges affecting quality of life and alterations in daily physiological processes persist. Among the health dimensions most affected are sleep quality and circadian rhythms, resulting in significant biological alterations. The main objective of this study is to analyze the existing scientific evidence regarding the effects of HIV on sleep quality and on the alignment and synchronization of circadian rhythms. The bidirectional interaction between these physiological alterations and associated clinical, immunological, and metabolic factors in people living with HIV (PLHIV) was evaluated, emphasizing their integration. For this review, searches were conducted in the PubMed, Scopus, and Web of Science databases. Eligibility criteria were applied, resulting in a final selection of 15 studies based on search strategies. The findings demonstrated a high prevalence of poor sleep quality as well as delayed circadian phase. These chronobiological alterations were closely associated with systemic inflammation, altered melatonin levels, impaired cognitive performance, and an altered metabolic profile involving leptin, ghrelin, adiponectin, and peroxisome proliferator-activated receptor (PPAR) signaling. Despite the consistency of the results, the methodologies of individual studies varied, and a notable absence of clinical trials aimed at addressing poor sleep quality and chronodisruption was observed. A promising clinical approach involves complementing antiretroviral therapy (ART) with chronotherapeutic interventions and sleep hygiene. In conclusion, HIV can disrupt personal temporal organization at a systemic level; consequently, incorporating assessments of sleep and circadian rhythms into clinical care may be warranted, while chronotherapeutic interventions should be further investigated.

## 1. Introduction

### 1.1. HIV, Sleep, and Circadian Rhythms

Human immunodeficiency virus (HIV) is a retrovirus transmitted through body fluids that primarily targets the immune system, specifically CD4+ T lymphocytes [1]. It enters these cells by fusing with their membrane via fusion proteins, among which glycoprotein 120 (gp120) mediates initial binding to cellular receptors and co-receptors (CD4 and CCR5/CXCR4), while gp41 is the glycoprotein responsible for the fusion of the viral membrane with the cellular membrane [1]. Once inside, it can integrate into the host genome and persist in a latent state, or, under appropriate conditions, assemble into new viral particles [2]. Identified in the early 1980s, HIV infection became a global public health priority due to its high mortality rate and rapid progression to acquired immunodeficiency syndrome (AIDS) [3]. Initial management focused primarily on patient survival and viral load control. Over recent decades, combination antiretroviral therapy (ART) has transformed HIV from a fatal diagnosis into a manageable chronic condition, particularly in regions with higher sociodemographic status. Today, people living with HIV (PLHIV) can achieve a life expectancy comparable to that of the general population, though global prevalence remained high at an estimated 36.65 million cases in 2019 [3].

Increased survival has shifted clinical focus toward the long-term health and quality of life of PLHIV. A broad array of non-AIDS comorbidities has emerged, including cardiovascular disease, metabolic disorders, cognitive decline, chronic inflammation, and sleep disturbances [4,5]. These comorbidities persist because ART inhibits active viral replication but does not eradicate the integrated viral DNA within the host genome [2], which remains the primary barrier to a complete cure. Viral proteins such as Tat can induce viral replication above a certain threshold, thereby interrupting viral latency.

Sleep is a fundamental physiological process vital for health. In the general population, poor sleep quality correlates with increased risks of chronic illness, cognitive decline, and mortality [6]. In PLHIV, these dynamics are further complicated by interactions between the virus itself, ART regimens, and comorbid conditions such as sleep-disordered breathing (SDB) [7]. Poor sleep quality frequently goes undiagnosed in clinical practice due to a lack of standardized diagnostic criteria, compromising patient well-being.

Multiple studies report that 50% to 70% of PLHIV experience sleep disturbances and circadian rhythm disruptions [7,8], rates markedly higher than those observed in HIV-negative controls. These issues encompass insomnia, sleep fragmentation, excessive daytime sleepiness, and SDB. Such disruptions can impair immune function and cognitive performance [9], even when viral load is suppressed.

Circadian rhythms regulate the sleep–wake cycle and a wide range of physiological processes; however, they are frequently disrupted in PLHIV, with alterations in melatonin secretion and delays in circadian phase [10,11,12], even in individuals who maintain apparently regular rest schedules. Under healthy conditions, alignment between internal physiological processes and the external light–dark cycle is essential for homeostasis. Circadian rhythms also modulate immune function; for example, dendritic cells migrate rhythmically to lymph nodes under the control of central clock genes such as BMAL1 and inflammatory cytokines such as TNF-α [13], while inflammatory markers themselves exhibit daily rhythmicity [14].

Recent evidence further indicates that cell-associated HIV RNA exhibits circadian rhythmicity in PLHIV receiving ART [15]. Objective assessment techniques, including polysomnography [16], actigraphy [10], and dim light melatonin onset (DLMO) assessment [11], have enabled these sleep and circadian disturbances to be quantified. The high prevalence of sleep disorders in this population highlights the need to understand how viral factors, persistent inflammation, central nervous system alterations, and ART may affect sleep regulation and overall well-being [8,17,18]. This is clinically relevant because impaired sleep quality, including frequent nocturnal awakenings assessed using instruments such as the Pittsburgh Sleep Quality Index (PSQI), negatively affects health outcomes [5,8].

### 1.2. Clinical and Biological Relevance

Human physiology is synchronized across 24-h cycles by a central circadian clock located in the suprachiasmatic nucleus (SCN) of the anterior hypothalamus. The SCN receives light input via intrinsically photosensitive retinal ganglion cells expressing melanopsin, which are primarily sensitive to blue light [13]. Light signals synchronize the SCN to the external environment, allowing it to coordinate peripheral clocks located in the liver, adipose tissue, muscle, gut, and immune cells. This system regulates sleep timing, hormone secretion, metabolic processes, and immune responses.

Melatonin, which is secreted by the pineal gland, serves as an indirect marker of SCN function and internal temporal alignment. Its secretion increases during the evening and is suppressed by light exposure, particularly blue light. Melatonin connects circadian regulation with systemic immune and metabolic modulation. Dim light melatonin onset (DLMO) represents the gold standard for determining circadian phase in chronobiological research [11].

Circadian misalignment occurs when internal physiology and behavioral rest–activity patterns fall out of phase with environmental cycles. In PLHIV, this misalignment may stem from insufficient light exposure, viral protein effects on the central nervous system [15], ART-related metabolic alterations [5], social stress [19], sleep-disordered breathing [7,16], or intrinsic circadian instability.

Non-rapid eye movement (NREM) slow-wave sleep and rapid eye movement (REM) sleep facilitate metabolic clearance and synaptic homeostasis in the brain. Consequently, chronic sleep disruption contributes to cognitive deficits [9]. Sleepiness and daytime activity levels remain tightly linked to nocturnal sleep quality and underlying circadian integrity [9,17,20].

At the molecular level, circadian rhythms are generated through autoregulatory transcription–translation feedback loops. The CLOCK and BMAL1 heterodimer activates the transcription of Period (PER1/2/3) and Cryptochrome (CRY1/2) genes. As PER and CRY proteins accumulate, they translocate to the nucleus to inhibit CLOCK:BMAL1, creating ~24-h oscillations. These molecular clocks regulate metabolic pathways, immune responses, and viral transcription [13,15].

Sleep macroarchitecture consists of distinct NREM and REM stages. NREM includes N1 (light sleep), N2 (deeper sleep characterized by K-complexes and sleep spindles), and N3 (slow-wave sleep [SWS], dominated by high-amplitude delta waves responsible for physical restoration). REM sleep is critical for memory consolidation and emotional regulation, and healthy sleep architecture typically comprises four to six alternating NREM–REM cycles per night. Poor sleep quality refers to the subjective perception or quantitative impairment of sleep continuity and restorative quality, whereas sleep disorders represent specific clinical conditions, such as chronic insomnia or obstructive sleep apnea [8].

Sleep–wake rhythms represent behavioral patterns of activity and rest that can be objectively quantified by actigraphy using parameters such as M10, L5/L6, and interdaily stability (IS), whereas delayed circadian phase refers to a shift in circadian timing toward later hours, measurable through rest–activity timing and physiological markers such as DLMO [11,12]. At the microstructural level, sleep architecture encompasses micro-arousals and stage transitions that reflect sleep continuity [16].

Sleep-disordered breathing (SDB), including obstructive sleep apnea (OSA), involves upper airway collapse or inadequate ventilation during sleep, leading to intermittent hypoxia and recurrent micro-arousals, particularly during REM sleep when muscle tone decreases [16]. In parallel, despite suppressive ART, low-level viral transcription and viral persistence contribute to chronic immune activation. Viral replication and cytokine production are associated with persistent elevations of pro-inflammatory mediators, including IL-6, TNF-α, and C-reactive protein (CRP), with the latter directly correlating with sleep fragmentation and circadian phase delays [17].

Sleep regulation is also closely interconnected with metabolic pathways involving hormones such as leptin, ghrelin, and adiponectin, as well as peroxisome proliferator-activated receptors (PPAR-α and PPAR-γ). Leptin signals satiety, whereas ghrelin promotes hunger, and sleep restriction typically lowers leptin and increases ghrelin, thereby promoting caloric intake. Adiponectin exerts anti-inflammatory effects and enhances insulin sensitivity, while PPARs regulate lipid metabolism, insulin sensitivity, and clock gene expression. Furthermore, single nucleotide polymorphisms (SNPs) in genes such as ADIPOQ, LEP, GHRL, and PPARA have been linked to sleep disruption in PLHIV [18].

### 1.3. Rationale and Objectives of the Systematic Review

However, the available evidence remains fragmented across domains including sleep, chronobiology, metabolism, inflammation, and cognition [9,12,18]. Therefore, this review integrates these areas into a unified framework to clarify how HIV affects sleep and circadian organization while exploring potential chronotherapeutic approaches to improve patient care.

Within this context, the present systematic review examines sleep quality and circadian rhythms in PLHIV by synthesizing subjective, objective, and chronobiological data. The general objective of this review was to analyze and synthesize the existing scientific evidence regarding the effects of HIV on sleep quality and the alignment and synchronization of circadian rhythms. Specifically, the review aimed to characterize HIV-related alterations in subjective and objective sleep quality; identify alterations in circadian phase, amplitude, and stability; analyze the relationships between sleep and circadian disruptions and immune activation, metabolic parameters, and cognitive function; and evaluate the methodological quality and risk of bias of the included studies.

Consistent with these objectives and the predefined eligibility criteria, the review addressed the following research questions:

1. What alterations in subjective and objective sleep quality and circadian rhythms have been reported in PLHIV?
2. When comparative data is available, how do subjective and objective sleep quality and circadian measures in PLHIV differ from those observed in HIV-negative controls?
3. What relationships have been reported between sleep and circadian alterations and chronic inflammation, immune activation, cognitive function, and metabolic pathways in PLHIV?
4. What evidence exists regarding interventions designed to improve sleep quality and circadian synchronization in PLHIV?

## 2. Methods

### 2.1. Study Design, Search Strategy, and Databases

A systematic search was conducted in PubMed, Scopus, and Web of Science to identify studies examining sleep quality and circadian rhythms in people living with HIV (PLHIV). The search strategy combined terms related to HIV, sleep, and circadian rhythms using Boolean operators (AND and OR). The search was restricted to studies involving human adults, published in English, and appearing in first- or second-quartile (Q1/Q2) journals (see Appendix A, Table A1).

To strengthen methodological rigor and ensure a consistent minimum standard of peer-review and editorial scrutiny, eligibility was restricted to original studies published in journals ranked in the Q1/Q2 categories of the Journal Citation Reports (JCRs) at the time of publication. We acknowledge that journal quartile is not a direct indicator of the methodological quality of individual studies and that this criterion may introduce selection bias. Therefore, the Q1/Q2 restriction was used as an additional eligibility criterion rather than as a surrogate for study quality. Methodological robustness and internal validity were independently assessed for each included study using appropriate risk-of-bias tools. This combined approach aimed to increase the consistency and reliability of the evidence included in the review while maintaining an explicit and reproducible selection framework.

The literature search was conducted across all databases on 15 December 2025. Duplicate articles were removed using the Mendeley bibliographic manager. Additionally, this manager was used for the screening process (title/abstract and full text), and data extraction was performed independently by two reviewers, with discrepancies resolved through consensus with a third reviewer. We analyzed whether the selected studies reported associations with sleep quality and circadian rhythms in PLHIV and performed a descriptive analysis and data extraction of the main study characteristics of the studies between 15 January and 1 May 2026 (described in Table A3; see Appendix A).

This manuscript was registered on 8 August 2026, in the Open Science Framework (OSF) database (https://doi.org/10.17605/OSF.IO/KUHM3).

Out of 94 initially identified records, 15 studies met all eligibility criteria and were included in the final synthesis. The distribution across databases was 10 results for PubMed, 22 results for Scopus, and 62 results for Web of Science.

### 2.2. Categorization of Studies by Thematic Blocks

1. Subjective and Clinical Sleep: [5,7,8].
2. Objective Sleep (Actigraphy/Polysomnography): [4,16,18,21].
3. Physiological Circadian Rhythms: [10,11,12,20].
4. Cognition and Social Rhythms: [9,17,19].
5. Molecular Clock Mechanisms and HIV: [15].

### 2.3. Methodological Quality Assessment and Risk of Bias

The risk of bias was assessed using the Risk Of Bias In Non-randomized Studies of Interventions (ROBINS-I) tool [22], covering 7 domains (D1: Confounding, D2: Selection of participants, D3: Classification of interventions/exposures, D4: Deviations from intended interventions, D5: Missing data, D6: Measurement of outcomes, D7: Selection of reported results). Overall judgments were categorized as: 🟢 Low, 🟡 Moderate, or 🔴 High risk of bias (see Appendix A, Table A2).

### 2.4. Eligibility Criteria

Eligible studies were those published between 2015 and 2025 that included adults (≥18 years) living with HIV, used randomized controlled, quasi-experimental, cohort, or cross-sectional designs, were published in English, and explicitly assessed sleep quality or circadian parameters. Exclusion criteria comprised systematic, meta-analytic, and narrative reviews; animal or strictly in vitro studies, except for preclinical molecular clock studies contextualizing viral transcription; severe HIV-associated neurocognitive disorders or structural CNS lesions; active substance dependence; regular night-shift work during the previous 3–6 months; recent transmeridian travel; and studies that did not report ART status or viral load parameters.

## 3. Results

**Figure 1:**
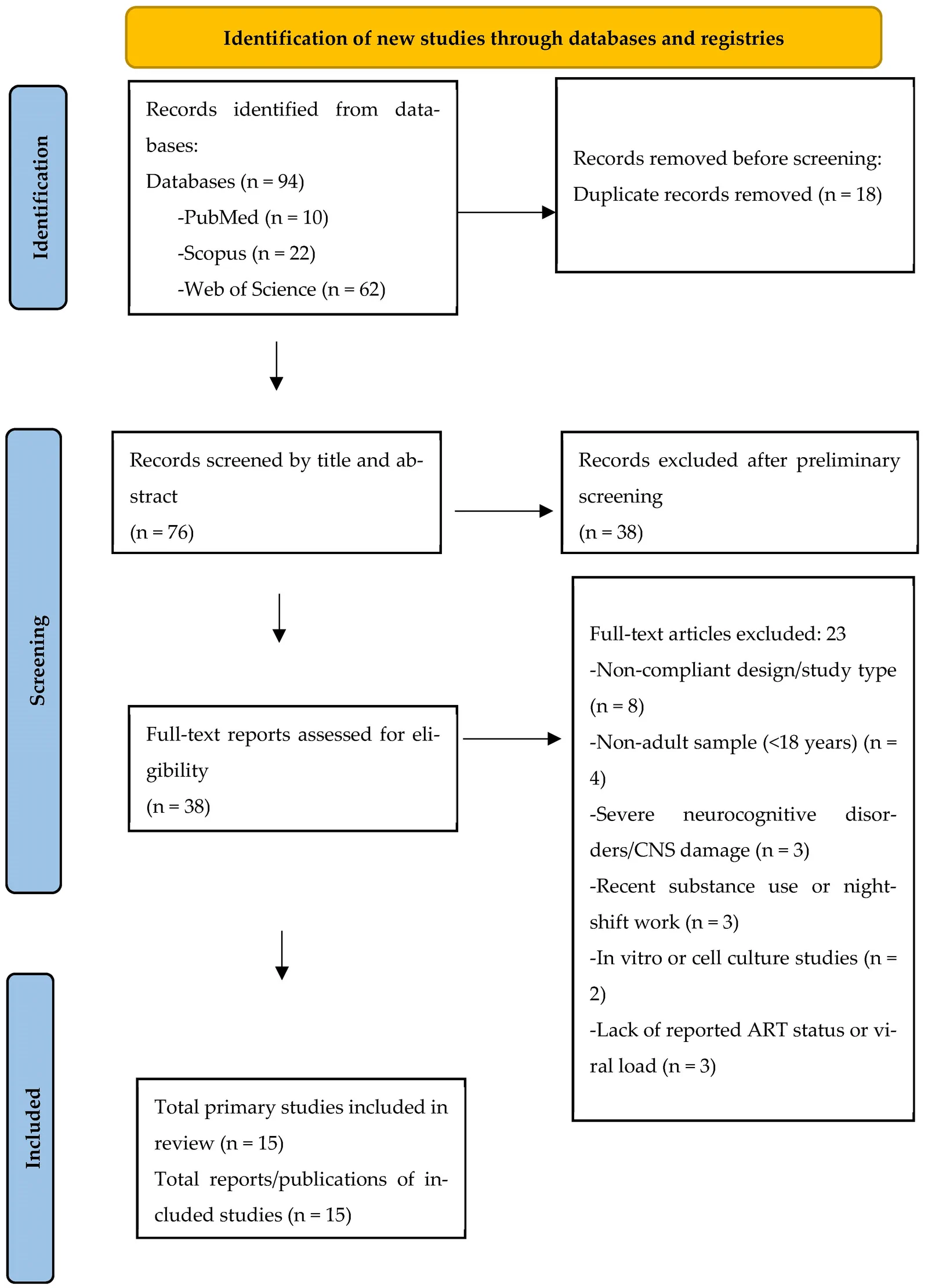
PRISMA 2020 flowchart for database searches in the PubMed, Scopus, and Web of Science databases [23] (Supplementary Materials).

Analysis of the included studies identified a convergence of findings across several domains, including subjective sleep quality, objective sleep measures, sleep architecture, circadian rhythms, inflammation, metabolic processes, and molecular mechanisms. Overall, the evidence suggests that PLHIV exhibit alterations in both sleep and circadian organization. These findings support the presence of interconnected sleep and chronobiological disturbances, although the predominantly observational nature of the evidence limits causal interpretation. The study selection process and the final inclusion of the 15 eligible studies are summarized in Figure 1.

### 3.1. Subjective and Objective Sleep Quality

Across subjective questionnaire studies using the PSQI, high rates of poor sleep quality (PSQI > 5) were consistently observed in PLHIV (>60%) [8]. Modern integrase strand transfer inhibitor (INSTI)-based ART regimens generally showed no negative impact on sleep compared to non-INSTI regimens [7]. However, older regimens containing central nervous system-active agents such as Efavirenz were associated with sleep disturbances and neuropsychological symptoms [24]. Switching to newer agents like bictegravir was associated with improved sleep quality [7]. Furthermore, untreated PLHIV not receiving ART exhibited even worse sleep outcomes [11].

Polysomnographic (PSG) evaluations revealed that sleep-disordered breathing (SDB) is a major contributor to sleep impairment in PLHIV [16]. PLHIV with severe SDB (>30 events/hour) exhibited significantly increased WASO, prolonged N1 light-sleep stage, and reduced NREM-to-REM stage transitions. Total sleep duration did not differ significantly between virally suppressed PLHIV without SDB and HIV-negative controls, but sleep continuity was compromised [18].

Actigraphic monitoring over 14-day periods showed lower interdaily stability (IS), reduced relative amplitude (RA), and increased night-to-night variability in PLHIV [12]. Accelerometry data from 1209 participants showed that PLHIV spent more time in a recumbent position and exhibited lower levels of daytime physical activity during the M10 period [20]. Detectable viral loads were associated with an additional 53 min of recumbent rest per day [10]. Additionally, sleep onset occurred later, and wake times differed from those of controls [3].

### 3.2. Circadian Rhythms and Inflammation

PLHIV exhibited marked circadian phase delays and diminished rhythm robustness. DLMO testing revealed a significant delay of over 1 h in evening melatonin secretion onset in PLHIV compared to controls [11]. Actigraphy confirmed corresponding delays (~40 min) in sleep midpoint and activity acrophase [12].

These chronobiological phase delays correlated with higher systemic inflammation, specifically elevated C-reactive protein (CRP) levels [17]. Furthermore, while total 24-h urinary 6-sulfatoxymelatonin output did not differ significantly between women with and without HIV, lower melatonin excretion was associated with lower sleep efficiency [10].

### 3.3. Daytime Sleepiness and Cognitive Function

Excessive daytime sleepiness and frequent daytime naps were significantly associated with poorer cognitive performance and slower processing speed in middle-aged and older PLHIV. Circadian phase delays measured by actigraphy also correlated with slower reaction times [9].

### 3.4. Metabolic and Genetic Factors Associated with Sleep and Circadian Rhythms

Genetic variations in energy homeostasis pathways were associated with sleep phenotypes in PLHIV. Specific single nucleotide polymorphisms (SNPs) in *LEP*, *ADIPOQ*, *GHRL*, and *PPARA* were associated with altered sleep duration and increased sleep fragmentation (WASO). Higher BMI was associated with poorer subjective sleep scores, while circulating adiponectin was associated with reduced WASO, and leptin and ghrelin levels were related to total sleep time (TST) [18]. At the molecular level, circadian regulation was also observed in HIV transcription. Cell-associated HIV RNA in CD4+ T cells from virally suppressed individuals exhibited endogenous 24-h rhythmicity [15], supporting an association between HIV transcriptional activity and circadian organization.

### 3.5. Treatments to Improve Sleep in People Living with HIV

Combined sleep hygiene interventions and low-dorse trazodone improved sleep and cognitive scores in PLHIV undergoing methadone maintenance [25]. Metabolomic profiling identified dysregulation in the tryptophan–kynurenine pathway as an objective correlate of actigraphic sleep disruption [21,26].

## 4. Discussion

The main objective of this review was to analyze the existing scientific evidence on the effects of HIV on sleep quality and on the alignment and synchronization of circadian rhythms. The main findings are discussed and interpreted in relation to the existing literature.

This review provides a unified physiological framework linking sleep architecture, circadian alignment, immune activation, and metabolic signaling in PLHIV. The synthesized findings indicate that PLHIV experience high rates of sleep fragmentation, daytime fatigue, circadian phase delay, and reduced rhythm amplitude. These disruptions persist even under effective ART and viral suppression. Rather than being solely behavioral, these alterations may be associated with underlying biological mechanisms involving persistent low-grade inflammation (elevated CRP, IL-6), altered CNS signaling, and metabolic gene variants (LEP, ADIPOQ, PPARA) [17,18]. Discrepancies exist regarding total sleep time (TST). While PSG and actigraphy studies in high-income cohorts showed similar TST between suppressed PLHIV and controls [12,16], rural cohort studies reported shortened TST in older PLHIV [4]. Methodological variations, environmental light exposure, and age differences likely explain these discrepancies.

Regarding melatonin, nocturnal urinary excretion totals were similar between groups [8], but the timing of secretion (DLMO) was significantly delayed [11], confirming that HIV impacts circadian timing rather than absolute hormonal production capacity.

Untreated HIV infection and detectable viral loads were associated with greater daily recumbent time, lower physical activity, and higher sleep fragmentation [20]. Although suppressive ART mitigates severe deterioration, low-level viral transcription and chronic inflammation maintain mild-to-moderate chronodisruption [3,12,25].

Evidence regarding therapeutic interventions specifically targeting sleep and circadian disturbances in PLHIV remains limited. Changes in ART regimens, particularly switching from efavirenz-based therapy, have been associated with improvements in sleep-related and neuropsychological outcomes [24]. Behavioral sleep interventions combined with low-dose trazodone have also shown improvements in sleep and cognitive outcomes in PLHIV receiving methadone maintenance therapy [25]. Furthermore, alterations in the tryptophan–kynurenine pathway have been associated with objectively measured sleep disruption [21]; however, these findings represent mechanistic evidence rather than demonstrated therapeutic efficacy. Overall, the available evidence highlights potential therapeutic targets but remains insufficient to establish specific chronotherapeutic strategies.

This review combines subjective, objective, physiological, and molecular evidence, providing a comprehensive perspective on sleep and circadian alterations in PLHIV. A major strength is the inclusion of rigorous objective assessment methods, including polysomnography (PSG), actigraphy, dim light melatonin onset (DLMO), and quantitative polymerase chain reaction (qPCR). Additionally, the included studies encompass geographically and demographically diverse cohorts, broadening the scope of the evidence considered. Nevertheless, several limitations should be acknowledged. The available evidence is predominantly based on observational and non-randomized studies, with a scarcity of large-scale randomized controlled trials specifically addressing circadian alignment and the molecular mechanisms underlying chronodisruption. Furthermore, restricting the literature search to studies published in Q1/Q2 journals was intended to ensure a high baseline of peer-reviewed methodological rigor; however, journal quartiles do not directly reflect the methodological quality of individual studies, and this criterion may have excluded relevant evidence published in lower-quartile journals, potentially introducing selection bias. Finally, residual confounding related to psychiatric comorbidities, social zeitgebers, and other individual or environmental factors may influence the observed associations between HIV, sleep, and circadian rhythms.

Clinicians should routinely screen PLHIV for sleep disorders and SDB using validated tools and actigraphy when appropriate. Interventions should combine sleep hygiene education, optimization of ART regimens, management of SDB, and social rhythm stabilization. Future research should prioritize longitudinal cohort studies and clinical trials testing targeted chronotherapies (e.g., timed light therapy, exogenous melatonin, etc.).

Accurately characterizing chronodisruption in people living with HIV (PLHIV) requires evolving from traditional static measurements toward continuous and integrated digital phenotyping models. Emerging trends point to medical-grade wearables equipped with photoplethysmography sensors, continuous ambient light photometry, and peripheral body temperature sensing, combined with continuous glucose monitoring (CGM) and continuous non-invasive echocardiography. Integrating these high-resolution temporal signals using deep learning algorithms and generative artificial intelligence may facilitate the development of chronobiological digital twins. Such models may eventually help predict fluctuations in viral latency, inflammatory activity, or metabolic risk and could support more individualized chronotherapeutic strategies.

## 5. Conclusions

The sleep and circadian rhythm disturbances identified in this review may have important implications for the quality of life of people living with HIV (PLHIV).

HIV is associated with poorer sleep quality, characterized by an increase in insomnia symptoms, greater sleep fragmentation, reduced sleep efficiency, and poorer subjective sleep perception.

These findings are confirmed by both subjective measures and objective records, reinforcing the consistency of the evidence. The results show that HIV also affects the organization of circadian rhythms, generating alterations in the phase, amplitude, and stability of biological rhythms. Phase delays, sleep–wake cycle desynchronization, and changes in physiological markers such as melatonin secretion are identified, indicating a disruption of the circadian system.

The findings support a close association between sleep disturbances and circadian dysregulation in PLHIV, potentially reflecting the interaction of several mechanisms. These include persistent systemic inflammation, alterations in melatonin secretion, metabolic changes, and the possible direct influence of the virus on circadian regulation. Furthermore, the effects of ART on sleep and circadian rhythms appear to depend on the specific antiretroviral agent rather than on ART as a therapeutic class.

Regarding the methodological quality of the evidence, most studies present a moderate risk of bias, especially due to residual confounding and the frequent use of subjective sleep measures.

Cross-sectional studies show greater limitations in establishing causal relationships, while cohort studies and experimental designs provide more robust evidence, although they remain subject to certain methodological limitations. In this regard, sleep and circadian disruptions should be recognized as clinically relevant features in people living with HIV in the era of effective antiretroviral therapy.

The need for research, evaluation, and treatments focused on regulating sleep and circadian synchronization is evident, with the goal of improving the long-term quality of life and well-being of people living with HIV.

## Data Availability

The data presented in this study are openly available in [Open Science Framework (OSF)] [10.17605/OSF.IO/KUHM3]. Data will be made available upon request by emailing the first author.

## Supplementary Materials

The following supporting information can be downloaded at: https://www.mdpi.com/article/doi/s1.

## Abbreviations

The following abbreviations are used in this manuscript:

ART	Antiretroviral Therapy
BMAL1	Brain and Muscle ARNT-Like 1
CD4	Cluster of Differentiation 4
CLOCK	Circadian Locomotor Output Cycles Kaput
CRP	C-reactive protein
CRY	Cryptochrome
DLMO	Dim Light Melatonin Onset
DSB	Daytime Sleep Behavior
IL-6	Interleukin 6
BMI	Body Mass Index
IS	Interdaily Stability
IV	Intradaily Variability
LTR	Long Terminal Repeat
L5/L6	Hours of lowest daily activity
M10	10 h of highest daily activity
NREM	Non-Rapid Eye Movement
OSF	Open Science Framework
PER	Period gene
PLHIV	People Living With HIV
PPAR	Peroxisome Proliferator-Activated Receptor
PSG	Polysomnography
PSQI	Pittsburgh Sleep Quality Index
qPCR	Quantitative Polymerase Chain Reaction
RA	Relative Amplitude
REM	Rapid Eye Movement
SCN	Suprachiasmatic Nucleus
SDB	Sleep-Disordered Breathing
SIK	Salt-Inducible Kinases
SWS	Slow Wave Sleep
TNF-α	Tumor Necrosis Factor alpha
TST	Total Sleep Time
HIV	Human Immunodeficiency Virus
WASO	Wake After Sleep Onset

## Appendix A

**Table A1:** Search strategies in the PubMed, Scopus and Web of Science databases.

Database	Syntax and Search String	Applied Filters and Limiters	Results
**PubMed**	(“HIV”[Mesh] OR “HIV Infections”[Mesh] OR “HIV”[Title/Abstract] OR “Human Immunodeficiency Virus”[Title/Abstract] OR “PLHIV”[Title/Abstract] OR “People living with HIV”[Title/Abstract]). AND (“Sleep”[Mesh] OR “Sleep Quality”[Mesh] OR “Circadian Rhythm”[Mesh] OR “Circadian Clocks”[Mesh] OR “sleep”[Title/Abstract] OR “sleep quality”[Title/Abstract] OR “circadian rhythm”[Title/Abstract] OR “circadian disruption”[Title/Abstract] OR “chronodisruption”[Title/Abstract] OR “phase delay”[Title/Abstract]).	• Publication date: 2015–2025 • Species: Humans • Age: Adult (≥19 years) • Language: English • Search fields: MeSH & Title/Abstract	10
**Scopus**	TITLE-ABS-KEY ((“HIV” OR “Human Immunodeficiency Virus” OR “PLHIV” OR “People living with HIV”) AND (“Sleep” OR “Sleep Quality” OR “Circadian Rhythm*” OR “Circadian Clock*” OR “Circadian Disruption” OR “Chronodisruption” OR “Phase Delay”)) AND PUBYEAR > 2014 AND PUBYEAR < 2026 AND (LIMIT-TO (DOCTYPE, “ar”)) AND (LIMIT-TO (LANGUAGE, “English”)).	• Publication years: 2015–2025 • Document type: Research articles (“ar”) • Language: English • Fields: TITLE-ABS-KEY	22
**Web of Science**	TS = (“HIV” OR “Human Immunodeficiency Virus” OR “PLHIV” OR “People living with HIV”) AND TS = (“Sleep” OR “Sleep Quality” OR “Circadian Rhythm*” OR “Circadian Clock*” OR “Circadian Disruption” OR “Chronodisruption” OR “Phase Delay”).	• Publication years: 2015–2025 • Document type: Journal Articles • Language: English • Field: Topic (TS) • Quality: Q1/Q2-indexed (JCRs)	62

Note. HIV, Human Immunodeficiency Virus; JCRs, Journal Citation Reports; MeSH, Medical Subject Headings; PLHIV, People Living With HIV; TITLE-ABS-KEY, Title, Abstract, Keywords search field in Scopus; TS, Topic Search field in Web of Science.

**Table A2:** Risk-of-Bias Assessment of the Included Studies Using the ROBINS-I Tool.

Study	D1	D2	D3	D4	D5	D6	D7	Quality/Overall Judgment
Gómez-Olivé et al. [4]	🟡	🟡	🟡	🟡	🟢	🟡	🟡	🟡
Aouizerat et al. [18]	🟡	🟢	🟡	🟢	🟢	🟢	🟢	🟡
Yoshino et al. [5]	🟡	🟢	🟡	🟡	🟢	🔴	🟡	🟡
Stern et al. [15]	🟢	🟢	🟢	🟢	🟢	🟢	🟢	🟢
Li et al. [9]	🟡	🟢	🟡	🟡	🟢	🟡	🟡	🟡
Rogando et al. [21]	🟡	🟢	🟡	🟡	🟢	🟢	🟢	🟡
Etzkorn et al. [20]	🟡	🟢	🟡	🟡	🟢	🟢	🟡	🟡
Bruno et al. [8]	🟡	🟢	🟡	🟡	🟢	🔴	🟡	🟡
Meng et al. [19]	🔴	🟢	🟡	🟡	🟢	🔴	🟡	🔴
Redman et al. [11]	🟡	🟢	🟡	🟢	🟢	🟢	🟢	🟡
Burgess et al. [10]	🟡	🟢	🟡	🟢	🟢	🟢	🟢	🟡
Lam et al. [7]	🟡	🟡	🟡	🟡	🟢	🟡	🟡	🟡
Punjabi et al. [16]	🟡	🟢	🟡	🟢	🟢	🟢	🟡	🟡
Borker et al. [12]	🟡	🟢	🟡	🟡	🟢	🟢	🟡	🟡
Haghayegh et al. [17]	🟡	🟢	🟡	🟡	🟢	🟡	🟡	🟡

Note. D1, bias due to confounding; D2, bias in participant selection; D3, bias in exposure classification; D4, bias due to intervention deviations; D5, bias due to incomplete data; D6, bias in outcome measurement; D7, bias in the selection of reported outcomes. 🟢 Low, 🟡 Moderate, or 🔴 High risk of bias. The 15 studies presented in this table constitute the studies included in the systematic review. Reference numbers in brackets correspond to their citations in the reference list and allow them to be distinguished from background and methodological references cited elsewhere in the manuscript.

**Table A3:** Description and extraction of the main characteristics of the studies.

Authors	Year	Country	Objective	Design	Participants	Tools/Measures	Main Findings
Gómez-Olivé et al. [4]	2018	South Africa	Analyze sleep, comorbidities, and mortality	Cohort study	1600 adults (≥45 years) with/without HIV	Actigraphy	Short sleep duration is linked to higher mortality
Aouizerat et al. [18]	2021	USA	Analyze metabolic genetics and sleep	Cross-sectional	289 PLHIV	Actigraphy & genotyping	Variants in *LEP*, *ADIPOQ*, and *GHRL* are associated with sleep fragmentation
Yoshino et al. [5]	2022	Japan	Analyze the relationship between sleep quality and HIV complications	Cross-sectional	1031 PLHIV	PSQI	High prevalence of poor sleep quality
Stern et al. [15]	2022	USA	Evaluate the rhythmicity of viral RNA	Longitudinal observational	20 male PLHIV on ART	Serial qPCR	Viral RNA exhibits a 24-h circadian rhythmicity
Li et al. [9]	2022	China	Evaluate daytime sleepiness and cognitive function	Cross-sectional	536 adults	DSB questionnaires & cognitive batteries	Daytime sleepiness is linked to worse executive function
Rogando et al. [21]	2022	USA	Link sleep to tryptophan–kynurenine pathway	Longitudinal observational	301 female PLHIV	Actigraphy & metabolomics	Pathway activation is associated with objective sleep fragmentation
Etzkorn et al. [20]	2022	USA	Analyze physical activity rhythms	Cohort study	1209 males (MACS cohort)	M10, L6, RA	Lower daytime activity and higher recumbent time in PLHIV
Bruno et al. [8]	2023	Italy	Assess prevalence of poor sleep quality	Cross-sectional	397 PLHIV	PSQI	>60% prevalence of poor sleep quality
Meng et al. [19]	2023	China	Analyze sleep, social rhythms, and depression	Cross-sectional	489 PLHIV	Questionnaires	Irregular social rhythms negatively affect sleep quality
Redman et al. [11]	2023	South Africa	Evaluate circadian phase via DLMO	Quasi-experimental	27 PLHIV, 27 controls	DLMO & Actigraphy	Circadian phase delayed by >1 h in PLHIV
Burgess et al. [10]	2023	USA	Analyze overnight melatonin	Cohort study	316 women with/without HIV	Urinary ELISA	Lower melatonin levels correlate with worse sleep efficiency
Lam et al. [7]	2024	USA	Evaluate clinical sleep disorders	Cohort study	~9000 PLHIV	Clinical diagnoses	High clinical prevalence of insomnia
Punjabi et al. [16]	2025	USA	Analyze sleep architecture	Cohort study	447 PLHIV, 349 controls	Polysomnography	Higher N1 sleep and lower sleep efficiency in PLHIV
Borker et al. [12]	2025	USA	Analyze rest–activity rhythms	Longitudinal observational	126 PLHIV, 55 controls	Actigraphy	Rest–activity rhythms are delayed and less robust in PLHIV
Haghayegh et al. [17]	2025	USA	Link circadian phase, inflammation, and cognition	Longitudinal observational	100 PLHIV	Actigraphy & inflammatory markers	Circadian delay strongly correlates with CRP and cognitive slowing

Note. ART, Antiretroviral Therapy; PLHIV, People Living With HIV; PSQI, Pittsburgh Sleep Quality Index. The 15 studies presented in this table constitute the studies included in the systematic review. Reference numbers in brackets correspond to their citations in the reference list and allow them to be distinguished from background and methodological references cited elsewhere in the manuscript.
